# Characterization of the Abracl-Expressing Cell Populations in the Embryonic Mammalian Telencephalon

**DOI:** 10.3390/biom13091337

**Published:** 2023-08-31

**Authors:** Dimitrios Troumpoukis, Andreas Rafail Vasileiou, Nikistratos Siskos, Electra Stylianopoulou, Petros Ypsilantis, George Skavdis, Maria E. Grigoriou

**Affiliations:** 1 Laboratory of Developmental Biology & Molecular Neurobiology, Department of Molecular Biology & Genetics, Democritus University of Thrace, GR-681 00 Alexandroupolis, Greeceilstylian@mbg.duth.gr (E.S.); 2 Laboratory of Molecular Regulation & Diagnostic Technology, Department of Molecular Biology & Genetics, Democritus University of Thrace, GR-681 00 Alexandroupolis, Greece; gskavdis@mbg.duth.gr; 3 Laboratory of Experimental Surgery and Surgical Research, Department of Medicine, Democritus University of Thrace, GR-681 00 Alexandroupolis, Greece

**Keywords:** Abracl, forebrain, development, immunofluorescence, progenitors, interneurons, fiber tracts

## Abstract

Abracl (ABRA C-terminal-like protein) is a small, non-typical winged-helix protein that shares similarity with the C-terminal domain of the protein ABRA (Actin-Binding Rho-Activating protein). The role of Abracl in the cell remains elusive, although in cancer cells, it has been implicated in proliferation, migration and actin dynamics. Our previous study showed that *Abracl* mRNA was expressed in the dividing cells of the subpallial subventricular zone (SVZ), in the developing cortical plate (CP), and in the diencephalic SVZ; however, the molecular identities of the Abracl-expressing cell populations were not defined in that work. In this study, we use double immunofluorescence to characterize the expression of Abracl on sections of embryonic murine (E11.5-E18.5) and feline (E30/31-E33/34) telencephalon; to this end, we use a battery of well-known molecular markers of cycling (Ki67, Ascl1, Dlx2) or post-mitotic (Tubb3, Gad65/67, Lhx6 and Tbr1) cells. Our experiments show that Abracl protein has, compared to the mRNA, a broader expression domain, including, apart from proliferating cells of the subpallial and diencephalic SVZ, post-mitotic cells occupying the subpallial and pallial mantle (including the CP), as well as subpallial-derived migrating interneurons. Interestingly, in late embryonic developmental stages, Abracl was also transiently detected in major telencephalic fiber tracts.

## 1. Introduction

Abracl (ABRA C-terminal-like protein, known also as Costars or HSPC280) is a low-molecular-weight (MW~9 kDa, 81 amino acid residues) protein that is found in all eukaryotes except fungi and shares similarity with the C-terminal, Actin-Binding Domain 2, (ABD2) of ABRA (Actin-Binding Rho-Activating protein) [1]. Abracl is a non-typical winged-helix protein with a tertiary structure consisting of three α-helices packed against an antiparallel β-sheet of four strands. However, it is unlikely that Abracl binds DNA in the same way as typical winged-helix proteins; the recognition helix α3 bears mostly negatively charged residues, while the hydrophilic groove that accommodates the DNA backbone in winged-helix DNA-binding proteins, is, in Abracl, hydrophobic [1].

Abracl was first identified as a protein expressed in several cell types of adult human tissues, as well as in cancer cells [1]. The gene encoding Abracl is located on mouse chromosome 10; human ABRACL is encoded by *c6orf115*, on chromosome 6.

Several studies have been performed to unravel the role of Abracl in various organisms, yet its role, so far, remains elusive. Costars, the Abracl homolog in *D. discoideum*, is required for cell motility upon chemoattractant stimuli [2]. In addition, human ABRACL has been implicated in cancer cell migration [3,4,5], probably by modulating actin dynamics [4]. Other works point to a role of Abracl in cell proliferation. Abracl promotes the proliferation of esophageal and breast cancer cell lines [3,5], and has been shown to be upregulated in cancer cells from patients with endometrial, bladder or gastric tumors [6,7,8]. Notably, in accordance with a role in proliferation, proteomic studies have shown that Abracl is present in the primary *cilium* [9]. Moreover, in *A. mexicanum*, Abracl is upregulated upon spinal cord regeneration [10], while in vitro experiments in the Neuro2A cell line have shown that its overexpression prevents differentiation and keeps the cells in a proliferative state [11].

We have previously analyzed *Abracl* expression during murine embryogenesis using in situ hybridization, and we showed that *Abracl* mRNA is highly expressed in the progenitor cells of the subventricular (SVZ) and mantle (M) zones of the subpallium, in the developing cortical plate (CP), as well as in the SVZ of the thalamus [11]. Notably, single-cell RNA sequencing analyses of E12.5 and E14.5 embryonic mouse forebrains showed similar results [12,13]; however, in these studies, expression of *Abracl* mRNA was also detected in migratory cell populations. In addition, these results, along with the emerging role of Abracl in proliferation, migration and invasion, possibly through regulation of actin dynamics [3,4,5,11], prompted us to study the molecular identity of Abracl-expressing populations within the embryonic forebrain. We show that, throughout embryogenesis, Abracl is detected in the dividing cells of the subpallial SVZ, as well as in the subpallial-derived migrating interneurons and in the post-mitotic neurons of the pallium. Moreover, in later stages of development, Abracl is highly expressed in major fiber tracts of the forebrain. Finally, this expression pattern is very well conserved between the murine and feline forebrain.

## 2. Materials and Methods

### 2.1. Animals

All experiments in mice were conducted in accordance with PD 160/92, in compliance with Directive 86/609/EEC, which was the legislation in force at the time of experimentation. The protocol was approved by the Animal Care and Use Committee of the Prefecture of Evros, Thrace, Greece, under the permit T/1571/13.5.09. Time-mated (*Mus musculus*) pregnant female mice (C57/BL6) were sacrificed at various stages of pregnancy (the day of vaginal plug detection was considered as E0.5) between E11.5 and E18.5.

Feline (*Felis catus*) embryos were obtained from clinically healthy domestic cats referred to the Unit of Obstetrics & Surgery of the Companion Animal Clinic of the School of Veterinary Medicine (Faculty of Health Sciences, Aristotle University of Thessaloniki) for preventive ovariohysterectomy. Staging of the feline embryos was performed as described in [14]. In brief, gestational age was initially assessed during the presurgical physical examination, as well as by macroscopic examination of the gravid uterus. After fixation, embryos were staged according to their morphology and crown–rump length [15,16]. Previous works have demonstrated that the first pallial neurons are generated in the feline telencephalon at around E30 [17,18,19]. To this end, we used feline fetuses around E30/31, which, according to a published prediction model, is roughly equivalent to the murine E13/14 [20]. Moreover, to visualize Abracl expression in fiber tracts in cats, we used E33/34 fetuses, which approximate E15 in the mouse, and present a well-developed internal capsule.

### 2.2. Tissue Preparation, Fixation and Sectioning

Feline embryos (at E30/31 and E34/35) and mouse embryos at E11.5, E12.5 and E13.5 were dissected free of maternal tissues in cold phosphate-buffered saline (PBS, pH7.4) and fixed in 4% *w*/*v* paraformaldehyde (PFA; in PBS) for at least 24 h at 4 °C. E15.5 and E18.5 mouse embryos were transcardially perfused with PBS (pH 7.4) followed by 4% *w*/*v* PFA; then, the brain was dissected and post-fixed in 4% *w*/*v* PFA for at least 24 h at 4 °C.

Following fixation, tissues were washed with PBS, cryoprotected in 30% *w*/*v* sucrose in PBS, embedded in Tissue Freezing Medium (Leica, Wetzlar, Germany) in the appropriate plane (coronal or sagittal) and stored at −80 °C until sectioning. Then, 12 μm sections were generated using a Leica CM1900UV cryostat, collected on Superfrost plus (Fisher Scientific, Waltham, MA, USA) slides, air dried for at least 30 min, and stored at −80 °C until later use.

### 2.3. Primary Cultures

For cultures of primary embryonic cells, E13.5 embryos were decapitated, and the subpallium was carefully dissected from the pallium in cold HBSS (Gibco, Billings, MT, USA) with 400 U/mL penicillin and 0.4 mg/mL streptomycin (PAN Biotech, Aidenbach, Germany). Following mechanical trituration, the cells were washed with HBSS with 400 U/mL penicillin and 0.4 mg/mL streptomycin, suspended in Neurobasal medium (Gibco) supplemented with 2% *v*/*v* B27 (Gibco), 2 mM Glutamine and 0.6% *w*/*v* glucose, plated on collagen-coated (Gibco) coverslips, and incubated at 37 °C in a humidified atmosphere with 5% *v*/*v* CO_2_. The following day, the cells were fixed with 4% *w*/*v* PFA, washed with PBS (3 washes, 5 min each) and with Tris-buffered saline (TBS, 3 washes, 5 min each), and either used immediately or stored at 4 °C for a maximum of 20 days.

### 2.4. Immunofluorescence

For immunofluorescence (IF), sections were post-fixed for 20 min with 4% *w*/*v* PFA. Heat-induced antigen retrieval was performed using 10 mM Sodium Citrate (pH 6) for 10 min in a microwave at nearly boiling temperature. Sections were allowed to cool down to room temperature (RT), washed in PBS for 5 min, permeabilized and blocked in 1% *w*/*v* bovine serum albumin (BSA), 0.3% *v*/*v* Triton-X in PBS for 1 h at RT and incubated overnight (O/N) at 4 °C with the primary antibodies diluted in 0.1% *w*/*v* BSA and 0.1% *v*/*v* Triton-X in PBS (Table 1). The next day, sections were washed in PBS (3 washes, 5 min each) and incubated for 1 h at RT with the secondary antibodies diluted in 0.1% *w*/*v* BSA and 0.1% *v*/*v* Triton-X in PBS (Table 2) with DAPI (Invitrogen, Carlsbad, CA, USA, 1:100). Sections were washed in PBS (3 washes, 5 min each) and mounted in 10% *w*/*v* Mowiol (Sigma, Burlington, MA, USA) and 25% *w*/*v* glycerol in 0.1 M Tris pH: 8.5.

Immunofluorescence on primary cultures was performed as previously described [21]. Briefly, cells were washed three times with TBS, permeabilized with 0.1% *v*/*v* Triton-X in TBS for 15 min, and incubated in blocking buffer (5% *v*/*v* goat serum in TBS) for 1 h at RT. Primary antibodies were diluted in 0.5% *v*/*v* goat serum in TBS (Table 1) and incubated O/N at 4 °C. Cells were washed three times with TBS for 5 min and incubated with the secondary antibodies diluted in 0.5% *v*/*v* goat serum in TBS (Table 2) for 1 h. Following three washes with TBS for 5 min, cells were incubated with DAPI (1:100) diluted in 0.5% *v*/*v* goat serum in TBS for 15 min and finally mounted onto slides using Mowiol solution. Experiments were performed three times using primary cells from different embryos. Approximately 1100 DAPI-positive cells were evaluated. The percentage of cells expressing Abracl as well as the percentage of cells expressing each marker were calculated. For each marker (and for Abracl) the arithmetic mean and standard deviation (SD) was calculated (Supplementary Tables S1–S3).

### 2.5. Microscope Imaging and Processing

Images were acquired with a Leica DM5500 B (Leica Microsystems) microscope equipped with a DFC7000T or a DFC310FX digital camera (Leica Microsystems) and captured using camera software (LAS v4.13, Leica Microsystems). For the experiments presented in Figure 4, image acquisition was performed on a customized Andor Revolution Spinning Disk Confocal system (Yokogawa CSUX1; Yokogawa, Tokyo, Japan), built around an Olympus IX81 (Olympus Shinjuku, Tokyo, Japan), with 60× 1.42NA oil lens (UPlanXApo; Olympus Shinjuku, Tokyo, Japan) and a digital camera (Andor Zyla 4.2 sCMOS; Andor Technology Ltd., Belfast, Northern Ireland). The system was controlled using Andor IQ3.6 software (Andor Technology). Images were acquired as z-stacks with a z-step of 1 μm. Images were processed in ImageJ version 2.9.0/1.53t (imagej.nih.gov). In young embryo brains (E11.5, E12.5 and E13.5), autofluorescence from blood vessels was removed digitally in Photoshop 2023 24.3.0 Release (Adobe) using layer masking. Cell count was performed using the ImageJ plugins “Colocalization Object Counter” and “Colocalization Image Creator” [22]. Schemata were created with the GIMP v.2.10 (gimp.org). Image panels were assembled in Photoshop 2023 24.3.0 Release (Adobe).

## 3. Results

In order to study Abracl expression during embryogenesis, we performed a series of double IF assays using anti-Abracl and a battery of antibodies against well-known molecular markers [Ki67, Ascl1, Dlx2 for cycling cells; Tubb3, Gad65/67 (abbreviated as Gad), Lhx6 and Tbr1 for post-mitotic cells]. IF assays were performed mostly on serial sections of various stages of murine (E11.5, E12.5, E13.5, E15.5 and E18.5) and, for corroboration purposes, feline (E30/31 and E34/35) embryos. Moreover, we used double IF assays on primary cultures of the E13.5 pallium and subpallium, in order to estimate the percentage of various cell populations.

### 3.1. In the Subpallium, Abracl Is Expressed by Proliferating and Post-Mitotic Cells

**Figure 1:**
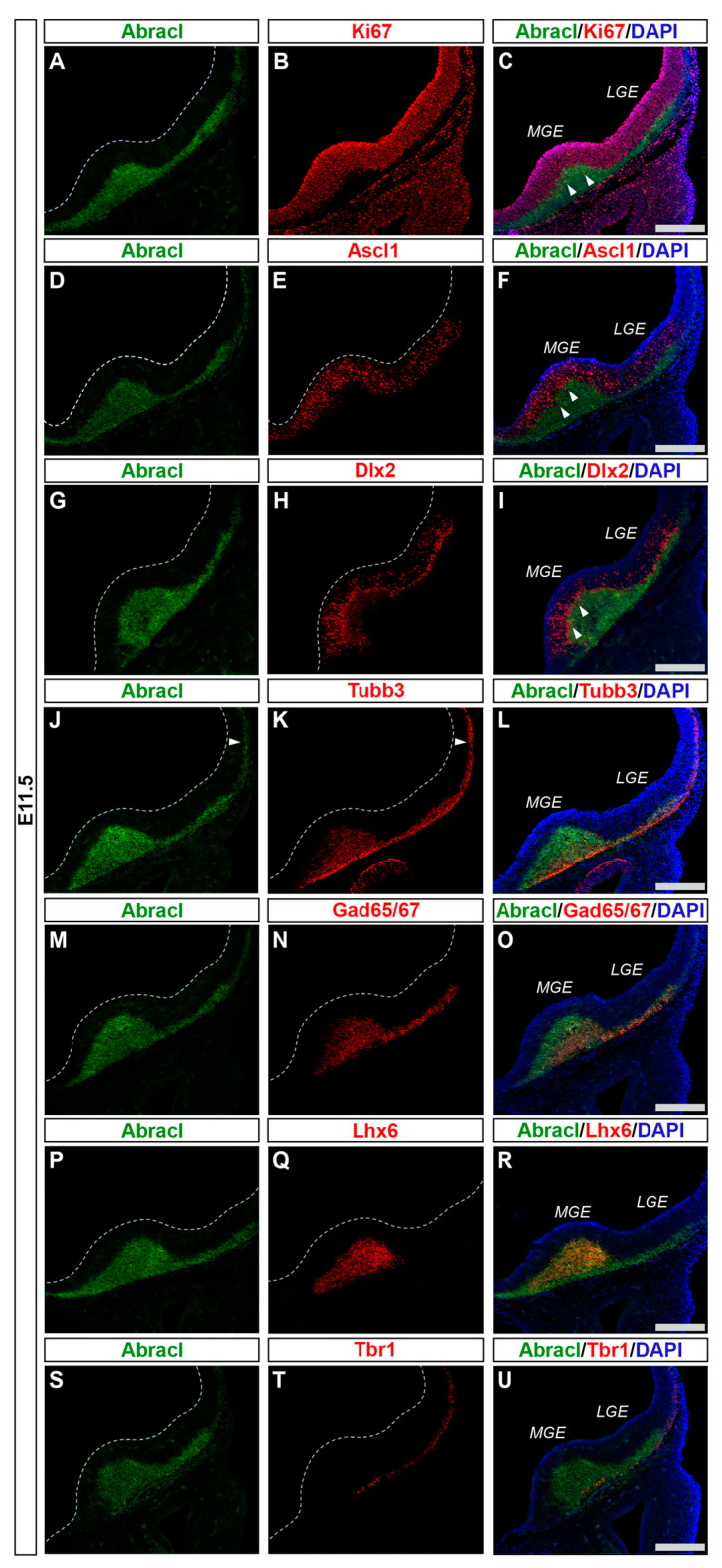
At E11.5, Abracl was highly expressed in the SVZ and the MZ of the subpallium. Double immunofluorescence on serial coronal sections of the E11.5 brain with antibodies for Abracl (**A**,**D**,**G**,**J**,**M**,**P**,**S**) and Ki67 (**B**), Ascl1 (**E**), Dlx2 (**H**), Tubb3 (**K**), Gad65/67 (**N**), Lhx6 (**Q**), and Tbr1 (**T**). Images in (**C**,**F**,**I**,**L**,**O**,**R**,**U**) are merged images, along with DAPI staining for the nuclei. Arrowheads in (**C**,**F**,**I**) point to cells positive for both Abracl and Ki67 (**C**), Ascl1 (**F**) and Dlx2 (**I**). Arrowheads in (**J**,**K**) indicate the expression of Abracl in post-mitotic cells of the pallium (compare Abracl and Tubb3). LGE, lateral ganglionic eminence; MGE, medial ganglionic eminence. Scale bars: 200 μm.

**Figure 2:**
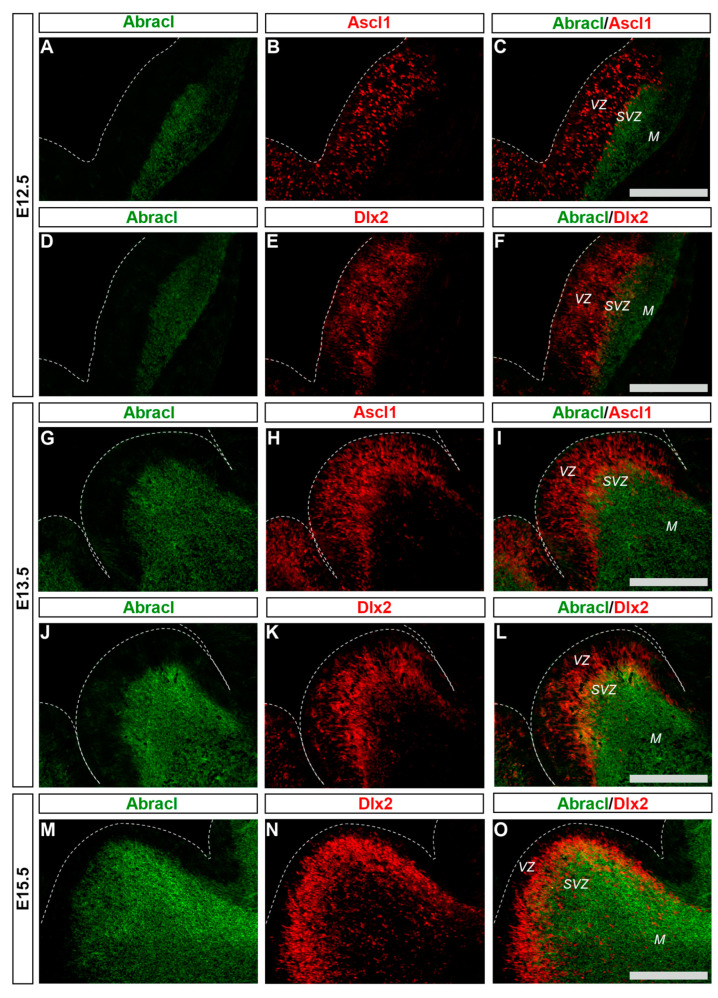
In the developing SVZ, Abracl is co-expressed with Ascl1 and Dlx2. Double immunofluorescence on coronal sections of E12.5 (**A**–**F**), E13.5 (**G**–**L**) and E15.5 (**M**–**O**) embryos for Abracl (**A**,**D**,**G**,**J**,**M**) and Ascl1 (**B**,**H**) or Dlx2 (**E**,**K**,**N**). The images in (**C**,**F**,**I**,**L**,**O**) are merged images. VZ, ventricular zone; SVZ, subventricular zone; M, mantle. Scale bars: 200 μm.

**Figure 3:**
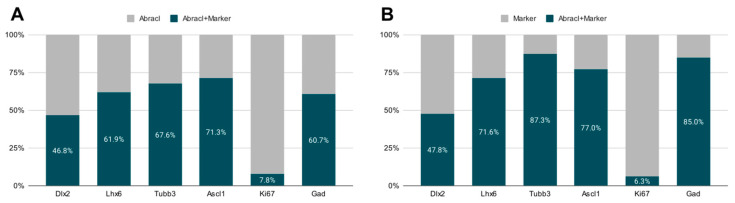
Quantitative analysis of double immunofluorescence experiments on E13.5 subpallial primary cultures with antibodies against Abracl and Dlx2, or Tubb3, Ascl1, Ki67, or Gad, and of E13.5 MGE primary cultures for Abracl and Lhx6. (**A**) The percentage of cells positive for both Abracl and each of the following markers: Dlx2, Lhx6, Tubb3, Ascl1, Ki67, and Gad. (**B**) The percentage of the cells positive for Abracl within the cell population that expresses each of the following markers: Dlx2, Lhx6, Tubb3, Ascl1, Ki67, and Gad.

**Figure 4:**
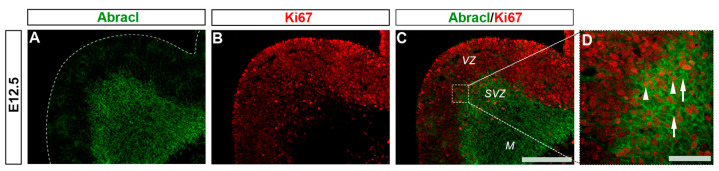
A small percentage of the Abracl-positive cells express Ki67. Double immunofluorescence on coronal sections of E12.5 embryos for Abracl (**A**) and Ki67 (**B**). The merged figure is also presented as captured through a wide field (**C**) or a confocal fluorescent microscope (**D**). Arrowheads in (**D**) indicate double-positive Abracl/Ki67 cells while arrows in (**D**) point to Abracl-positive/Ki67-negative cells. VZ, ventricular zone; SVZ, subventricular zone; M, mantle. Scale bars: 200 μm (**A**–**C**), 100 μm (**D**).

To analyze the expression of Abracl in the proliferative zones of the subpallium, antibodies against Ascl1, Dlx2 [23,24] and Ki67 were used in double IF experiments with an antibody against Abracl [11]. At E11.5, very few Abracl-expressing cells were positive for Ascl1 (arrowheads in Figure 1F) or Dlx2 (arrowheads in Figure 1I) in the emerging SVZ. At later stages, however, namely at E13.5 and at E15.5, the Ascl1-expressing domain within the SVZ presented as harboring many Abracl-positive cells (Figure 2A–C,G–I and Figure S2D–I); the analysis of E13.5 subpallial primary cultures revealed that 77.0% of the Ascl1-positive progenitors expressed Abracl, while 71.3% of the Abracl-expressing cells were also Ascl1 positive (Figure 3). Regarding Dlx2, a small number of double Abracl-/Dlx2-expressing cells could be observed within the SVZ as early as E11.5 (arrowheads in Figure 1I), while from E12.5 onwards, this population extended in a wider domain of the subpallial SVZ (Figure 2D–F,J–O and Figure S3); 47.8% of the Dlx2-expressing cells were also Abracl positive in the primary cultures (Figure 3). Within the SVZ, several cells co-expressed Ki67 with Abracl (white arrowheads in Figure 4D) at all stages studied, namely E13.5 (Figure S1D–F and Figure 3), E15.5 (Figure S1G–I), E18.5 (Figure S1J–L). Double IF analysis on primary cultures of E13.5 subpallium showed that 6.3% of the Ki67-expressing cells were Abracl positive and 7.8% of the Abracl-positive cells expressed Ki67 (Figure 3).

**Figure 5:**
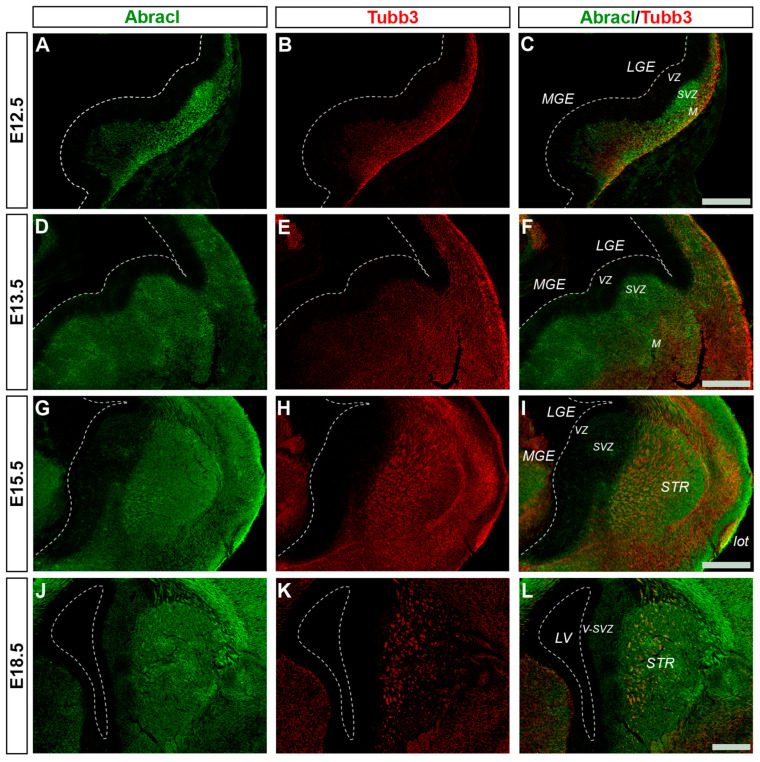
Abracl is broadly expressed in post-mitotic cells of the subpallium. Double immunofluorescence on coronal sections of E12.5 (**A**–**C**), E13.5 (**D**–**F**), E15.5 (**G**–**I**) and E18.5 (**J**–**L**) embryos for Abracl (**A**,**D**,**G**,**J**) and Tubb3 (**B**,**E**,**H**,**Κ**). The images in (**C**,**F**,**I**,**L**) are merged images. VZ, ventricular zone; SVZ, subventricular zone; M, mantle; STR, striatum; LV, lateral ventricle; lot, lateral olfactory tract; V-SVZ, ventricular–subventricular zone; LGE, lateral ganglionic eminence; MGE, medial ganglionic eminence. Scale bars: 200 μm.

Abracl was broadly expressed in the subpallial mantle; in fact, at E11.5, the majority of Abracl-expressing cells were observed within the Tubb3-positive domain (Figure 1J–L). In subsequent stages, E12.5–E18.5, the expression domain of Tubb3 in the MZ and the developing basal ganglia overlapped, to a large extent, with that of Abracl (Figure 5). At Ε13.5, double IF assays on primary cultures of the subpallium revealed that 87.3% of the Tubb3-expressing cells were Abracl positive and 67.6% of the Abracl-expressing cells were also positive for Tubb3 (Figure 3).

**Figure 6:**
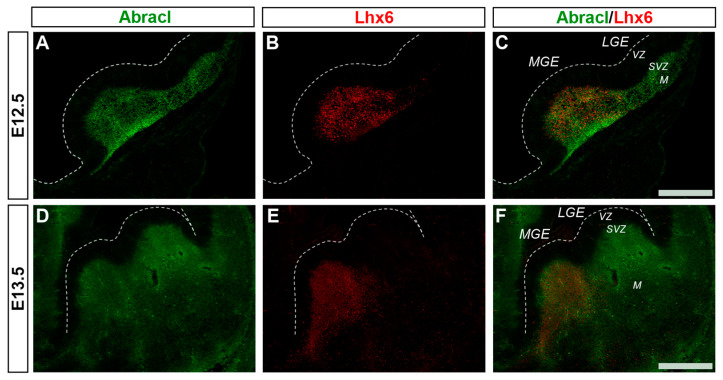
Abracl is expressed in immature MGE-derived interneurons, as revealed by double immunofluorescence with Lhx6. Double immunofluorescence on coronal sections of E12.5 (**A**–**C**) and E13.5 (**D**–**F**) embryos for Abracl (**A**,**D**) and Lhx6 (**B**,**E**). Images in (**C**,**F**) are merged images. LGE, lateral ganglionic eminence; MGE, medial ganglionic eminence; VZ, ventricular zone; SVZ, subventricular zone; M, mantle. Scale bars: 200 μm.

**Figure 7:**
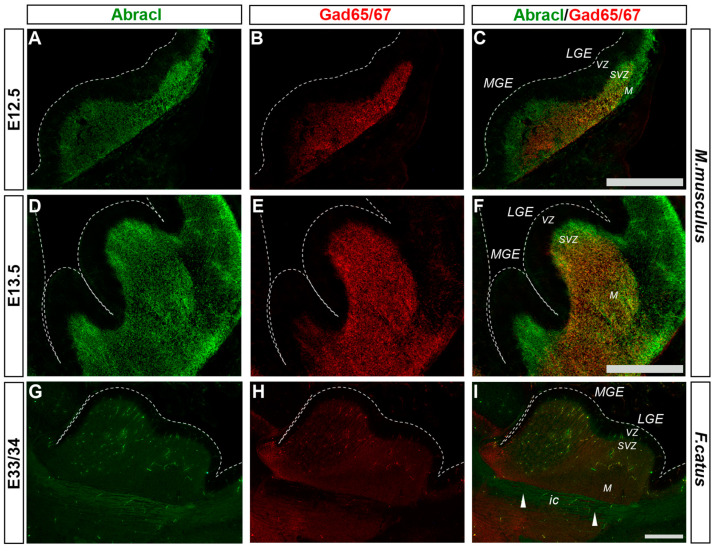
Abracl is expressed in immature GABAergic interneurons, as revealed by co-expression with Gad65/67. Double immunofluorescence on coronal murine brain sections of E12.5 (**A**–**C**) and E13.5 (**D**–**F**) embryos as well as sagittal sections of feline brains of E33/34 (**G**–**I**) embryos for Abracl (**A**,**D**,**G**) and Gad65/67 (**B**,**E**,**H**). Images in (**C**,**F**,**I**) are merged images. Arrowheads point to neuronal axons that cross the internal capsule and are Abracl positive. LGE, lateral ganglionic eminence; MGE, medial ganglionic eminence; VZ, ventricular zone; SVZ, subventricular zone; M, mantle; ic, internal capsule. Scale bars: 200 μm.

Cortical GABAergic interneurons, a highly heterogeneous group of neurons, are born in the subpallium and migrate tangentially to the pallium [25]. Lhx6 is expressed in the immature interneurons in the SVZ and MZ of the MGE during their tangential and radial migration into the cortex, as well as during their differentiation [26]. Notably, the expression domain of Lhx6 greatly overlapped with the expression domain of Abracl at all stages studied—E11.5 (Figure 1P–R), E12.5 (Figure 6A–C), and E13.5 (Figure 6D–F)—suggesting that Abracl is expressed in the immature cortical interneurons; additionally, Abracl was also detected in interneurons migrating to the pallium (see Section 3.2). Double IF experiments on MGE-derived primary cultures revealed that 71.6% of the Lhx6 population expressed Abracl and that 61.9% of the Abracl-expressing cells were positive for Lhx6 (Figure 3). Similar results were obtained in double IF experiments with Gad at E11.5 (Figure 1M–O), E12.5 (Figure 7A–C), and E13.5 (Figure 7D–F); in primary cultures of this stage, cells positive for Abracl cells accounted for up to 85% of the Gad population, while Gad-positive cells accounted for approximately 60.7% of the total Abracl-expressing cells (Figure 3). Abracl expression among the Gad-positive immature interneurons of the subpallial mantle was further corroborated on sections of E34/35 feline embryos (Figure 7G–I), arguing for a conserved pattern across mammals.

### 3.2. In the Pallium, Abracl Is Expressed Only by Post-Mitotic Cells

**Figure 8:**
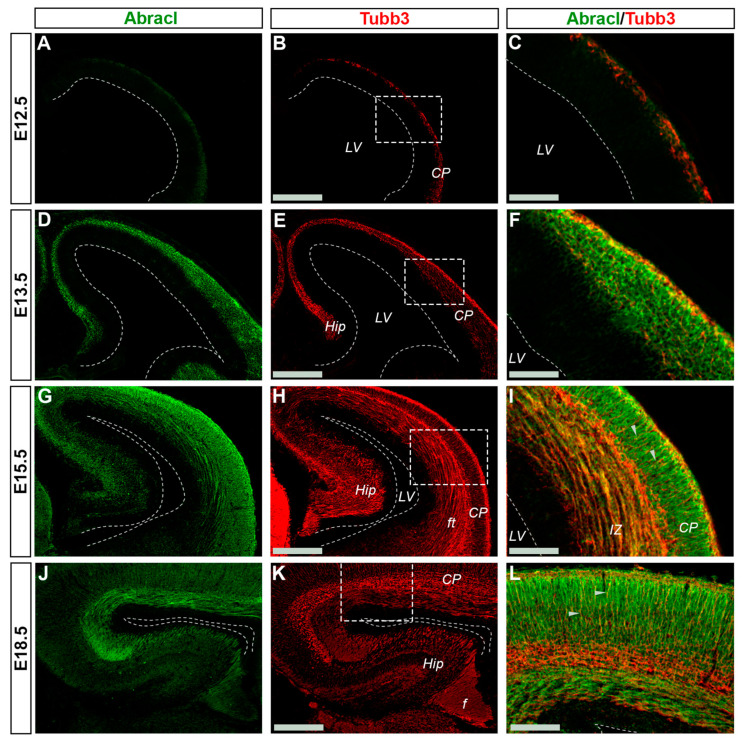
In the pallium Abracl is expressed by post-mitotic cells. Double immunofluorescence on coronal sections of E12.5 (**A**–**C**), E13.5 (**D**–**F**), E15.5 (**G**–**I**) and E18.5 (**J**–**L**) embryos for Abracl (**A**,**D**,**G**,**J**) and Tubb3 (**B**,**E**,**H**,**K**). Image in (**C**,**F**,**I**,**L**) are merged images of the boxed areas in (**B**,**E**,**H**,**K**). Arrowheads indicate intracortical projections that are positive for both Abracl and Tubb3. Note that Abracl is also co-expressed with Tubb3 in the fiber tracts and the fimbria. LV, lateral ventricle; CP, cortical plate; Hip, hippocampus; ft, fiber tracts; f, fimbria. Scale bars in (**B**,**E**,**H**,**K**): 200 μm; in (**C**,**F**,**I**): 90 μm; in (**L**): 60 μm.

**Figure 9:**
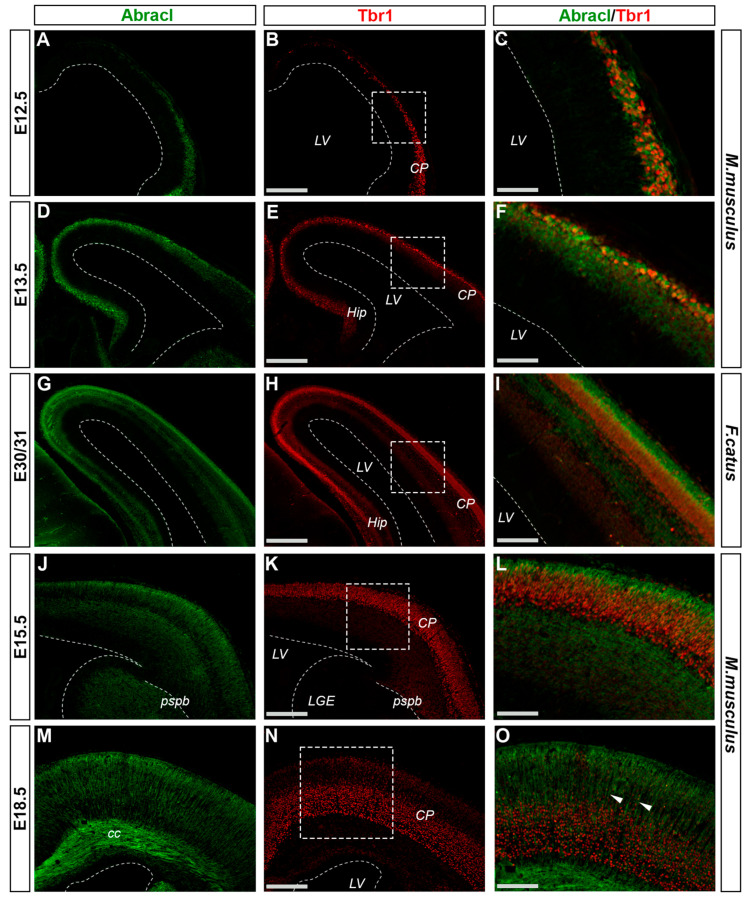
Abracl is detected in most of the cells in the developing cortical plate. Double immunofluorescence on coronal sections of E12.5 (**A**–**C**), E13.5 (**D**–**F**), E15.5 (**J**–**L**) and E18.5 (**M**–**O**) mouse embryos as well as E30/31 (**G**–**I**) feline embryos for Abracl (**A**,**D**,**G**,**J**,**M**) and Tbr1 (**B**,**E**,**H**,**K**,**N**). Images in (**C**,**F**,**I**,**L**,**O**) are merged images. Arrowheads indicate intracortical neuronal axons that are Abracl-positive. LV, lateral ventricle; CP, cortical plate; Hip, hippocampus; LGE, lateral ganglionic eminence; pspb, pallial–subpallial boundary; cc, corpus callosum. Scale bars in (**B**,**E**,**H**,**K**,**N**): 200 μm; in (**C**): 50 μm; in (**F**,**I**): 60 μm; in (**L**): 80 μm; in (**O**): 100 μm.

**Figure 10:**
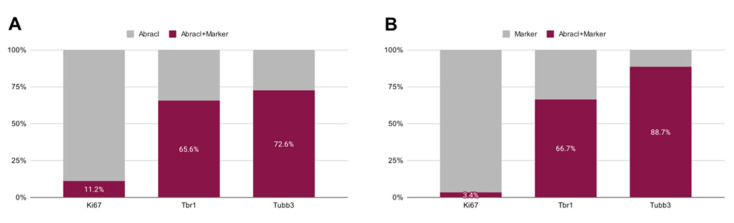
Quantitative analysis of the double immunofluorescence experiments on E13.5 pallial primary cultures with antibodies against Abracl and Ki67, or Tbr1 or Tubb3. (**A**) The percentages of cells positive for both Abracl and each one of the following markers: Ki67, Tbr1, Tubb3. (**B**) The percentages of the Abracl-expressing cells within the cell population that expresses each one of the following markers: Ki67, Tbr1, Tubb3.

In the pallium, Abracl expression was detected mainly in post-mitotic Tubb3-positive cells, at all stages examined, starting from E11.5 (arrowheads in Figure 1J,K) and lasting until the later stages of embryogenesis (Figure 8). Notably, when comparing Abracl expression domain with that of Tbr1, the former seems to be broader, while the latter is restricted within the cortical plate during the early (E12.5–13.5) stages (Figure 9A–F), and in the deep layers during the later (E15.5–18.5) stages (Figure 9J–O). Particularly from E15.5, Abracl expression appears to form a second superficial zone, yet minimally overlapping with that of Tbr1, serving as a marker of nascent superficial layers (Figure 9J–L). This finding was further corroborated in E30/31 feline embryos, suggesting a conserved trait in mammalian pallial neurogenesis (Figure 9G–I). Primary pallial cultures of E13.5 showed that 88.7% of the Tubb3-expressing cells were also Abracl-positive, while 66.7% of the Tbr1-positive cells expressed Abracl (Figure 10). Interestingly, double Ki67/Abracl IF experiments on sections did not reveal expression of Abracl in the pallial proliferative zones, at any stage examined (Figure S4). However, in E13.5 pallial primary cultures a small percentage of double Abracl/Ki67-expressing cells was detected: 3.4% of the Ki67-positive progenitors were Abracl positive, while 11.2% of the Abracl-positive cells expressed Ki67 (Figure 10). This population may represent cells that start expressing Abracl while in the process of leaving the cell cycle during, or immediately after, their last mitotic division.

**Figure 11:**
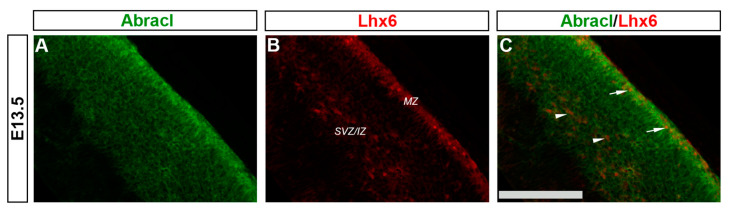
Abracl is expressed by immature migratory GABAergic interneurons. Double immunofluorescence on coronal sections of E13.5 embryos for Abracl (**A**) and Lhx6 (**B**). The image in (**C**) is a merged image. Arrowheads indicate double-positive cells in the SVZ/IZ migratory route while arrows point to double-positive cells in the MZ. SVZ/IZ, subventricular zone/intermediate zone; MZ, marginal zone. Scale bar: 110 μm.

**Figure 12:**
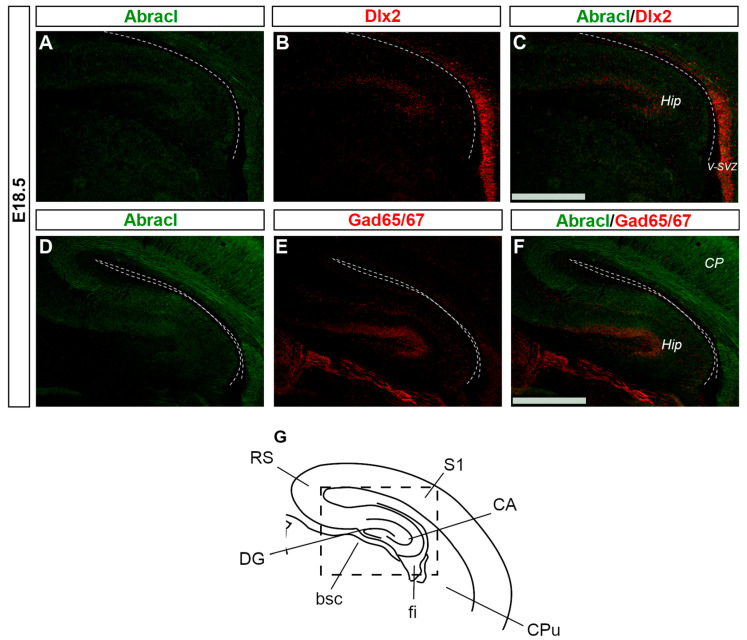
Abracl is expressed by migrating interneurons that reach the Hippocampus. Double immunofluorescence on coronal sections of E18.5 embryos for Abracl (**A**,**D**) and Dlx2 (**B**) or Gad65/67 (**E**). The images in (**C**,**F**) are merged images corresponding to the boxed area of the schematic representation (**G**). Dlx2 expression in the hippocampus at E18.5 starts to fade out; the high exposure used to detect hippocampal Dlx2 resulted in Dlx2 overexposure in the subpallial ventricular and subventricular zone. V-SVZ, ventricular–subventricular zone; CP, cortical plate; Hip, hippocampus; RS, retrosplenial cortex; S1, primary somatosensory cortex; CA, hippocampal CA fields; CPu, caudate putamen; fi, fimbria; bsc, brachium of the superior colliculus; DG, dentate gyrus. Scale bars: 200 μm.

In the pallium, double Abracl/Lhx6 IF experiments revealed that Abracl was expressed by subpallial-derived cortical interneurons migrating through the pallium. More specifically, the comparison between Abracl and Lhx6 expression patterns showed that double-positive cells were observed in both migration routes [25], namely, the pallial SVZ/IZ (white arrowheads in Figure 11C) and the pial surface (white arrows in Figure 11C). A closer examination of double Abracl/Dlx2 and Abracl/Gad IF results showed that Abracl was also present in subpallial-derived interneurons that reach the hippocampal primordium (Figure 12). These results show that Abracl is expressed in subpallial-derived interneurons that migrate tangentially and reach either the cortex or the hippocampus.

### 3.3. Abracl Is Expressed in Major Telencephalic Fiber Tracts

**Figure 13:**
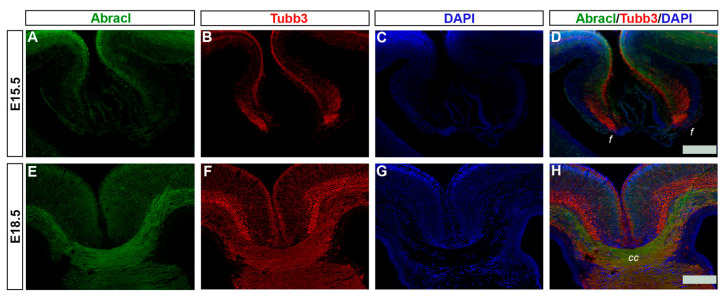
Abracl is expressed in developing neuronal fibers. Double immunofluorescence on coronal sections of E15.5 (**A**–**D**) and E18.5 (**E**–**H**) embryos for Abracl (**A**,**E**) and Tubb3 (**B**,**F**). (**C**,**G**) DAPI staining of nuclei; tracts are not stained as they lack nuclei, and therefore appear black. (**D**) and (**H**) are merged images. f, fimbria; cc, corpus callosum. Scale bars: 200 μm.

**Figure 14:**
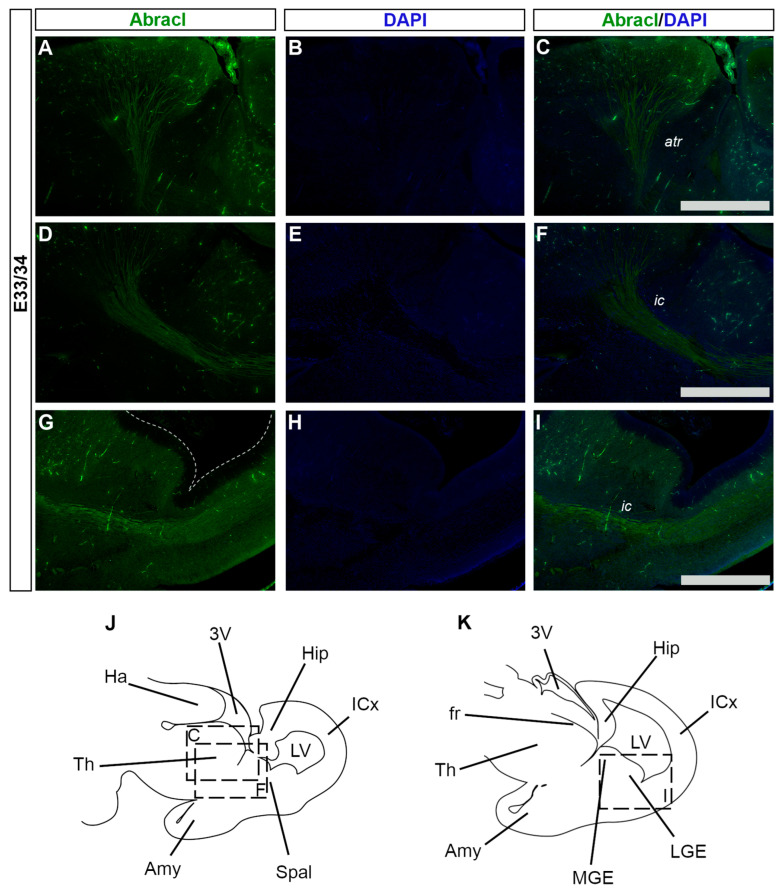
Abracl is expressed in developing feline neuronal fibers. Immunofluorescence on sagittal sections of E33/34 feline embryos for Abracl (**A**,**D**,**G**) followed by DAPI staining; tracts are not stained as they lack nuclei, and therefore appear black (**B**,**E**,**H**). Images (**A**–**C**) and (**D**–**F**) correspond respectively to the boxed areas “C” and “F” in the schematic representation (**J**). Images (**G**–**I**) correspond to the boxed area in the schematic representation (**K**). The images in (**C**,**F**,**I**) are merged images. atr, anterior thalamocortical radiation; ic, internal capsule; 3V, third ventricle; Hip, hippocampal formation; ICx, isocortical primordium; LV, lateral ventricle; Spal, subpallium; Amy, amygdala; Th, Thalamus; Ha, Habenula; fr, fasciculus retroflexus; MGE, medial ganglionic eminence; LGE, lateral ganglionic eminence. Scale bars: 200 μm.

In later stages of embryogenesis (E15.5 and E18.5), comparison of the Abracl expression domain with that of Tubb3, known to be dynamically expressed in neuronal fibers [27], showed that high levels of the former were detected in the major intracortical and corticofugal fiber tracts. As early as E15.5, Abracl expression was observed in associative (arrowheads in Figure 8I,L and Figure 9O) as well as commissural intracortical axons of the corpus callosum (cc, Figure 13), the anterior commissure (ac, Figure S6) and the fimbria (f, Figure 13 and Figure 8J,K). Abracl was also detected in corticofugal axons that cross the internal capsule (also part of the thalamocortical fibers that mediate the projection of cortical axons towards the diencephalon and *vice versa*) both in murine and feline embryos (Figure 14D–I, and Figure S5 and arrowheads in Figure 7I). Notably, at E18.5, Abracl expression in the pallium was observed mostly in neuronal axons and fiber tracts. Taken together, these results show that a population of post-mitotic pallial cells at the late stages of their differentiation upregulate Abracl expression also in the developing neuronal axons.

## 4. Discussion

**Figure 15:**
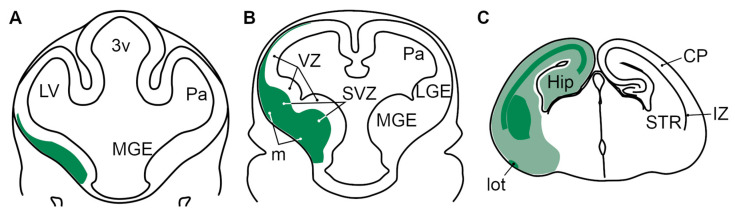
Schematic representation of the expression of Abracl in the telencephalon of E11.5 (**A**), E12.5 (**B**) and E18.5 (**C**) mouse embryos. At E11.5 (**A**) and E12.5 (**B**), Abracl (green) is expressed in the subpallial subventricular zone and mantle, as well as in the pallial subventricular zone, but not in the telencephalic ventricular zone. At E18.5 (**C**), Abracl is widely expressed in post-mitotic cells of the telencephalon (pale green); the developing striatum, as well as major telencephalic fiber tracts (like the lateral olfactory tract, or fiber tracts in the pallial intermediate zone) present higher levels of Abracl expression (dark green). 3v, third ventricle; LV, lateral ventricle; Pa, pallium; MGE, medial ganglionic eminence; LGE, lateral ganglionic eminence; VZ, ventricular zone; SVZ, subventricular zone; m, mantle; Hip, hippocampal primordium; lot, lateral olfactory tract; STR, striatum; CP, cortical plate; IZ, intermediate zone.

In the past 30 years, elegant studies using a variety of approaches have unraveled the principles implicated in the development of the forebrain [28,29,30,31,32]; however, our understanding of the cellular and molecular mechanisms underlying telencephalic neurogenesis and differentiation still remains limited. During mammalian embryogenesis, the expression of Abracl, a small and highly conserved protein present in all eukaryotes except fungi, is restricted in the developing forebrain; its role, however, remains elusive. Several recent publications have shown that, in vitro, in cancer cells lines, Abracl is implicated in the regulation of actin dynamics, and thus in proliferation, migration and invasion [3,4,5]. Expression data from our previous analysis of *Abracl* mRNA distribution [11], as well as from single-cell transcriptomics [12,13], delineated the expression pattern of *Abracl* mRNA without, however, characterizing the molecular identity of the *Abracl*-expressing cell populations, which would probably provide insight into its role in vivo during development. In this work, we show that (summarized in Figure 15), throughout embryogenesis, Abracl is highly expressed in post-mitotic cell populations of the pallium and the subpallium, as well as in dividing cells of the subpallial SVZ; in addition, it is expressed in the subpallial-derived migrating interneurons, as well as in major fiber tracts of the forebrain. Interestingly, the expression domain of Abracl protein is broader than that of the *Abracl* mRNA.

The SVZ of the subpallium appears around E11.5 in the MGE, and E12.5 in the LGE [33], and quickly increases in both size and number of dividing cells to become the main site of proliferation at E13.5–E14.5 [33,34,35]; this expansion is associated with an increase in the variety of the progenitor types found in the ganglionic eminences, which are related to the generation of different neuronal types [36]. High levels of Abracl were detected in the subpallial SVZ during neurogenesis; these results are in agreement with our previous analysis of the *Abracl* mRNA distribution [11]. In addition, they are supported by single-cell transcriptomic data, in which Abracl has been shown to be highly expressed in the subpallial SVZ of the E12.5 and E14.5 murine telencephalon [12,13]; in fact, Abracl was among the top five differentially expressed genes in the subpallial SVZ [13]. Our studies of the co-expression of Abracl with Dlx2, Ascl1 and Lhx6 [23,24,37,38,39] argue for a possible role of this protein in regulating SVZ neurogenesis and, therefore, cortical interneuron generation. At the peak of neurogenesis, the majority of Abracl-positive cells (71.3%) also express Ascl1; these Abracl-/Ascl1-positive cells could represent subapical progenitors (SAPs), outer radial glia (oRG) or intermediate progenitors (IPs; [40]). On the other hand, a small percentage of the subpallial SVZ co-expresses Abracl and Ki67, as revealed by IF experiments both on sections and on primary cultures; these cells are located at the SVZ–VZ border and could represent subapical progenitors (SAPs); more studies are, however, required to determine the exact nature of the Abracl-expressing progenitors within the subpallium.

Furthermore, Abracl was shown to be broadly expressed in the subpallial mantle, as revealed by double Abracl/Tubb3 and Abracl/Gad, either on sections or on dissociated cells. Interestingly, in our previous study, *Abracl* mRNA was not detected in the subpallial mantle [11]; we suggest that Abracl is transcribed and translated in the progenitors residing in the SVZ and is then inherited by their progeny of post-mitotic cells that migrate radially to the subpallial mantle. Notably, a recent study showed that, in cortical neuronal cells dissociated from E15.5 mouse embryos, Abracl has an extended half-life of approximately 189 h (for comparison, in the same study, the half-life of the bHLH transcription factor Neurod2 was estimated to be approximately 12 h) [41]. In other words, Abracl protein synthesized in the SVZ, at the peak of the mRNA expression at E13.5, may be present until birth, therefore supporting our hypothesis. An alternative scenario could be that *Abracl* mRNA is present in the subpallial mantle with expression levels below the detection limit of in situ hybridization.

In the subpallium, the broad spatial overlap between the Abracl expression domain and the domains of Ascl1, Dlx2, Lhx6 and Gad [23,24,37,38,39] suggests that Abracl plays a role in the generation of GABAergic neurons. Moreover, Abracl co-expression with Lhx6-positive neurons migrating through the pallial MZ and SVZ/IZ and reaching up to the hippocampus implies that it is also involved in the migration of subpallial-derived cortical interneurons [26]. Previous results have shown that *Abracl* mRNA can be detected within the cortical plate and the pallial SVZ (Figure 1D–O of [11]), an expression pattern that can be interpreted through the aforementioned prism of migrating interneurons. On the other hand, the present results, particularly the co-expression of Tbr1 and Abracl in pallial primary cultures, argue for a role in the development of cortical projection neurons. Furthermore, the analysis of double Abracl/Tbr1 IF assays in E15.5 and E18.5 murine as well as in E30/31 feline sections suggested a potential role for Abracl in the cortical plate and the generation of the upper cortical layers. Interestingly, Abracl was shown to be co-expressed with Ki67 in several cells from pallial primary cultures, suggesting a potential, yet disguised, role in pallial neurogenesis.

Given the similarity of Abracl to the ABD2 domain of ABRA, as well as its involvement in the modulation of actin dynamics in cancer cells [4], it is plausible that this protein may participate in the regulation of actin dynamics during development. Single-cell transcriptomics experiments point to such a role [12]. In addition, a high-throughput proteomics study in non-neuronal cell lines has shown that Abracl is associated with the primary cilia [9]. Given that primary cilia play a vital role in the neurogenic process [42], one could postulate that both *Abracl* mRNA and protein should be expressed in the VZ, where ciliated progenitors reside; our results show that neither *Abracl* mRNA nor Abracl were expressed in the VZ. As distinct molecular mechanisms regulate cell cycle progression and differentiation within VZ and SVZ (for instance the progenitors in the VZ use cyclin D1 while those in the SVZ cyclin D2; [43,44,45,46,47]), it is possible that the primary cilia structure is not identical in these zones. In addition, primary cilia play a role in regulating the migratory rhythm and the directionality of the cells in both radial and tangential migration, as well as in the reorientation from tangential to radial migration during the laminar allocation of the interneurons [48]. Therefore, in the migratory interneurons, Abracl could be involved in the migratory processes, possibly through its interaction with the cilia-assembling machinery.

Finally, our observation that Abracl is highly expressed in major telencephalic fiber tracts, especially in later prenatal stages, implies a role for this protein in axon elongation and/or synapse formation. The presence of Abracl along the fiber tracts may result either from local translation of the mRNA in the axonal or dendritic neuropil [49] or from active transport of the protein from the soma. A recent study of the hippocampal translatome in rats [50], however, showed a negative neuropil:soma ratio for Abracl, thus favoring the latter scenario (i.e., translation in the soma and transportation across the axon). Interestingly, this is in agreement with the fact that Abracl has a prolonged half-life. Regarding its function, the dynamic expression of Abracl in the fiber tracts of the murine and feline embryo, along with the findings about its implication in the regulation of actin dynamics [4], supports the hypothesis that Abracl is implicated in processes that depend on the actin cytoskeleton, such as axon growth, synapse formation and migration. Thus, transport along the axon following translation in the neuronal soma may be required for axonal elongation of the developing fiber tracts. Finally, our dual (in terms of species) analysis points out the conserved Abracl expression pattern between murine and feline embryo brains. Additionally, the absence of visible differences in the aforementioned pattern points to the conservation of the molecular mechanisms in which Abracl is implicated, at least up to the Boreoeutheria stem ancestor.

## Figures and Tables

**Table 1:** Primary antibodies used in this study.

Primary Antibody	Company	Catalog Number	Dilution
Rabbit anti-Abracl	[11]	-	1/100
Rabbit anti-Abracl	ATLAS Antibodies	HPA030217	1/100
Rat anti-Ki67	Invitrogen	14-5698-80	1/500
Mouse anti-Tubb3	Millipore	MAB1637	1/100 (1/1000 in cells)
Mouse anti-Dlx2	Santa Cruz antibodies	sc-393879	1/100
Mouse anti-Ascl1	Santa Cruz antibodies	sc-374104	1/100
Mouse anti-Gad65/67	Santa Cruz antibodies	sc-365180	1/100
Mouse anti-Tbr1	Proteintech	66564-1-Ig	1/100
Mouse anti-Lhx6	Santa Cruz antibodies	sc-271433	1/100

According to the manufacturer (Santa Cruz antibodies), anti-Gad65/67 recognizes the feline Gad65/67 protein. Anti-Tbr1 monoclonal antibody recognizes an epitope within the N-terminal 196 amino acid residues of the mouse protein. The feline Tbr1 is highly similar to the murine one (identity 99%), while protein BLAST did not reveal similarity to any other protein. The anti-ABRACL polyclonal antibody was raised against the murine ABRACL, which is highly similar to the feline one (96% identity), and protein BLAST did not reveal similarity to any other protein.

**Table 2:** Secondary antibodies used in this study.

Secondary Antibody	Company	Catalog Number	Dilution
Goat anti-rabbit IgG CF488A	Biotium	20015	1/2000
Donkey anti-rat IgG CF568	Biotium	20092-1	1/500
Donkey anti-mouse IgG CF568	Biotium	20105	1/500

## Data Availability

The original contributions presented in the study are included in the article/Supplementary Material, further inquiries can be directed to the corresponding authors.

## Supplementary Materials

The following supporting information can be downloaded at: https://www.mdpi.com/article/10.3390/biom13091337/s1, Figure S1: Abracl is expressed in the subpallial SVZ and mantle; Figure S2: Abracl is co-expressed with the neurogenic factor Ascl1 in the subpallial SVZ; Figure S3: Abracl is co-expressed with the interneuron lineage-specific transcription factor Dlx2 in the subpallial SVZ; Figure S4: Abracl is not expressed in proliferative zones of the pallium; Figure S5: Abracl is dynamically expressed in neuronal fibers; Figure S6: Abracl is dynamically expressed in neuronal fibers; Supplementary Table S1: Percentages of cells (DAPI-positive) expressing Abracl and respective markers; Supplementary Table S2: Percentages of Abracl-positive cells expressing each marker; Supplementary Table S3: Percentages of marker-positive cells that also express Abracl.

## References

[1] Lin J., Zhou T., Wang J. (2011). Solution Structure of the Human HSPC280 Protein: Solution Structure of HSPC280. Protein Sci..

[2] Pang T.-L., Chen F.-C., Weng Y.-L., Liao H.-C., Yi Y.-H., Ho C.-L., Lin C.-H., Chen M.-Y. (2010). Costars, a *Dictyostelium* Protein Similar to the C-Terminal Domain of STARS, Regulates the Actin Cytoskeleton and Motility. J. Cell Sci..

[3] Fan S., Chen P., Li S. (2021). miR-145-5p Inhibits the Proliferation, Migration and Invasion of Esophageal Carcinoma Cells by Targeting ABRACL. Biomed Res. Int..

[4] Hsiao B.-Y., Chen C.-H., Chi H.-Y., Yen P.-R., Yu Y.-Z., Lin C.-H., Pang T.-L., Lin W.-C., Li M.-L., Yeh Y.-C. (2021). Human Costars Family Protein ABRACL Modulates Actin Dynamics and Cell Migration and Associates with Tumorigenic Growth. Int. J. Mol. Sci..

[5] Li J., Chen H. (2022). Actin-Binding Rho Activating C-Terminal like (ABRACL) Transcriptionally Regulated by MYB Proto-Oncogene like 2 (MYBL2) Promotes the Proliferation, Invasion, Migration and Epithelial-Mesenchymal Transition of Breast Cancer Cells. Bioengineered.

[6] Ura B., Monasta L., Arrigoni G., Franchin C., Radillo O., Peterlunger I., Ricci G., Scrimin F. (2017). A Proteomic Approach for the Identification of Biomarkers in Endometrial Cancer Uterine Aspirate. Oncotarget.

[7] Chen Y., Xu T., Xie F., Wang L., Liang Z., Li D., Liang Y., Zhao K., Qi X., Yang X. (2020). Evaluating the Biological Functions of the Prognostic Genes Identified by the Pathology Atlas in Bladder Cancer. Oncol. Rep..

[8] Wang D., Liu H., Ren C., Wang L. (2019). High Expression of *ABRACL* Is Associated with Tumorigenesis and Affects Clinical Outcome in Gastric Cancer. Genet. Test. Mol. Biomark..

[9] Gupta G.D., Coyaud É., Gonçalves J., Mojarad B.A., Liu Y., Wu Q., Gheiratmand L., Comartin D., Tkach J.M., Cheung S.W.T. (2015). A Dynamic Protein Interaction Landscape of the Human Centrosome-Cilium Interface. Cell.

[10] Monaghan J.R., Walker J.A., Page R.B., Putta S., Beachy C.K., Voss S.R. (2006). Early Gene Expression during Natural Spinal Cord Regeneration in the Salamander Ambystoma Mexicanum: Gene Expression and Spinal Cord Regeneration. J. Neurochem..

[11] Stylianopoulou E., Kalamakis G., Pitsiani M., Fysekis I., Ypsilantis P., Simopoulos C., Skavdis G., Grigoriou M.E. (2016). HSPC280, a Winged Helix Protein Expressed in the Subventricular Zone of the Developing Ganglionic Eminences, Inhibits Neuronal Differentiation. Histochem. Cell Biol..

[12] Loo L., Simon J.M., Xing L., McCoy E.S., Niehaus J.K., Guo J., Anton E.S., Zylka M.J. (2019). Single-Cell Transcriptomic Analysis of Mouse Neocortical Development. Nat. Commun..

[13] Lee D.R., Rhodes C., Mitra A., Zhang Y., Maric D., Dale R.K., Petros T.J. (2022). Transcriptional Heterogeneity of Ventricular Zone Cells in the Ganglionic Eminences of the Mouse Forebrain. eLife.

[14] Siskos N., Ververidis C., Skavdis G., Grigoriou M.E. (2021). Genoarchitectonic Compartmentalization of the Embryonic Telencephalon: Insights from the Domestic Cat. Front. Neuroanat..

[15] Knospe C. (2002). Periods and stages of the prenatal development of the domestic cat. Anat. Histol. Embryol..

[16] Evans H.E., Sack W.O. (1973). Prenatal development of domestic and laboratory mammals: Growth curves, external features and selected references. Anat. Histol. Embryol..

[17] Glatzle M., Hoops M., Kauffold J., Seeger J., Fietz S.A. (2017). Development of deep and upper neuronal layers in the domestic cat, sheep and pig neocortex. J. Vet. Med. Ser. C Anat. Histol. Embryol..

[18] Luskin M.B., Shatz C.J. (1985). Neurogenesis of the cat’s primary visual cortex. J. Comp. Neurol..

[19] Luskin M.B., Shatz C.J. (1985). Studies of the earliest generated cells of the cat’s visual cortex: Cogeneration of subplate and marginal zones. J. Neurosci..

[20] Workman A.D., Charvet C.J., Clancy B., Darlington R.B., Finlay B.L. (2013). Modeling transformations of neurodevelopmental sequences across mammalian species. J. Neurosci..

[21] Chytoudis-Peroudis C.C., Siskos N., Kalyviotis K., Fysekis I., Ypsilantis P., Simopoulos C., Skavdis G., Grigoriou M.E. (2018). Spatial Distribution of the Full-Length Members of the Grg Family during Embryonic Neurogenesis Reveals a “Grg-Mediated Repression Map” in the Mouse Telencephalon. PLoS ONE.

[22] Lunde A., Glover J.C. (2020). A Versatile Toolbox for Semi-Automatic Cell-by-Cell Object-Based Colocalization Analysis. Sci. Rep..

[23] Casarosa S., Fode C., Guillemot F. (1999). *Mash1* Regulates Neurogenesis in the Ventral Telencephalon. Development.

[24] Eisenstat D.D., Liu J.K., Mione M., Zhong W., Yu G., Anderson S.A., Ghattas I., Puelles L., Rubenstein J.L. (1999). DLX-1, DLX-2, and DLX-5 Expression Define Distinct Stages of Basal Forebrain Differentiation. J. Comp. Neurol..

[25] Lavdas A.A., Grigoriou M., Pachnis V., Parnavelas J.G. (1999). The Medial Ganglionic Eminence Gives Rise to a Population of Early Neurons in the Developing Cerebral Cortex. J. Neurosci..

[26] Liodis P., Denaxa M., Grigoriou M., Akufo-Addo C., Yanagawa Y., Pachnis V. (2007). *Lhx6* Activity Is Required for the Normal Migration and Specification of Cortical Interneuron Subtypes. J. Neurosci..

[27] Menezes J.R., Luskin M.B. (1994). Expression of Neuron-Specific Tubulin Defines a Novel Population in the Proliferative Layers of the Developing Telencephalon. J. Neurosci..

[28] Molnár Z., Métin C., Stoykova A., Tarabykin V., Price D.J., Francis F., Meyer G., Dehay C., Kennedy H. (2006). Comparative Aspects of Cerebral Cortical Development. Eur. J. Neurosci..

[29] Guillemot F. (2007). Cell Fate Specification in the Mammalian Telencephalon. Prog. Neurobiol..

[30] Urban N., Guillemot F. (2014). Neurogenesis in the Embryonic and Adult Brain: Same Regulators, Different Roles. Front. Cell. Neurosci..

[31] Hardwick L.J.A., Philpott A. (2014). Nervous Decision-Making: To Divide or Differentiate. Trends Genet..

[32] Villalba A., Götz M., Borrell V. (2021). The Regulation of Cortical Neurogenesis. Current Topics in Developmental Biology.

[33] Smart I.H. (1976). A Pilot Study of Cell Production by the Ganglionic Eminences of the Developing Mouse Brain. J. Anat..

[34] Fentress J.C., Stanfield B.B., Cowan W.M. (1981). Observations on the Development of the Striatum in Mice and Rats. Anat. Embryol..

[35] Pilz G.-A., Shitamukai A., Reillo I., Pacary E., Schwausch J., Stahl R., Ninkovic J., Snippert H.J., Clevers H., Godinho L. (2013). Amplification of Progenitors in the Mammalian Telencephalon Includes a New Radial Glial Cell Type. Nat. Commun..

[36] Ross M.E. (2011). Cell Cycle Regulation and Interneuron Production. Dev. Neurobiol..

[37] Yun K., Potter S., Rubenstein J.L.R. (2001). *Gsh2* and *Pax6* Play Complementary Roles in Dorsoventral Patterning of the Mammalian Telencephalon. Development.

[38] Grigoriou M., Tucker A.S., Sharpe P.T., Pachnis V. (1998). Expression and Regulation of *Lhx6* and *Lhx7*, a Novel Subfamily of LIM Homeodomain Encoding Genes, Suggests a Role in Mammalian Head Development. Development.

[39] Katarova Z., Sekerková G., Prodan S., Mugnaini E., Szabó G. (2000). Domain-Restricted Expression of Two Glutamic Acid Decarboxylase Genes in Midgestation Mouse Embryos. J. Comp. Neurol..

[40] Turrero García M., Harwell C.C. (2017). Radial Glia in the Ventral Telencephalon. FEBS Lett..

[41] Mathieson T., Franken H., Kosinski J., Kurzawa N., Zinn N., Sweetman G., Poeckel D., Ratnu V.S., Schramm M., Becher I. (2018). Systematic Analysis of Protein Turnover in Primary Cells. Nat. Commun..

[42] Hasenpusch-Theil K., Theil T. (2021). The Multifaceted Roles of Primary Cilia in the Development of the Cerebral Cortex. Front. Cell Dev. Biol..

[43] Doetsch F., Caillé I., Lim D.A., García-Verdugo J.M., Alvarez-Buylla A. (1999). Subventricular Zone Astrocytes Are Neural Stem Cells in the Adult Mammalian Brain. Cell.

[44] Glickstein S.B., Moore H., Slowinska B., Racchumi J., Suh M., Chuhma N., Ross M.E. (2007). Selective Cortical Interneuron and GABA Deficits in Cyclin D2-Null Mice. Development.

[45] Glickstein S.B., Monaghan J.A., Koeller H.B., Jones T.K., Ross M.E. (2009). Cyclin D2 Is Critical for Intermediate Progenitor Cell Proliferation in the Embryonic Cortex. J. Neurosci..

[46] Noctor S.C., Martínez-Cerdeño V., Ivic L., Kriegstein A.R. (2004). Cortical Neurons Arise in Symmetric and Asymmetric Division Zones and Migrate through Specific Phases. Nat. Neurosci..

[47] Martynoga B., Drechsel D., Guillemot F. (2012). Molecular Control of Neurogenesis: A View from the Mammalian Cerebral Cortex. Cold Spring Harb. Perspect. Biol..

[48] Stoufflet J., Caillé I. (2022). The Primary Cilium and Neuronal Migration. Cells.

[49] Spaulding E.L., Burgess R.W. (2017). Accumulating Evidence for Axonal Translation in Neuronal Homeostasis. Front. Neurosci..

[50] Glock C., Biever A., Tushev G., Nassim-Assir B., Kao A., Bartnik I., Tom Dieck S., Schuman E.M. (2021). The Translatome of Neuronal Cell Bodies, Dendrites, and Axons. Proc. Natl. Acad. Sci. USA.

