# Supplementary material for: Characterization of the Abracl-Expressing Cell Populations in the Embryonic Mammalian Telencephalon
Source: Biomolecules. 2023 Aug 31;13(9):1337. doi: 10.3390/biom13091337 (PMC10527439; doi:10.3390/biom13091337)
Supplement: Supplementary file 1 [file biomolecules-13-01337-s001.zip › Supplementary Figure Legends.pdf]

biomolecules
MDPI

Supplementary Figure Legends

Figure S1. Abracl is expressed in the subpallial SVZ and M. Double immunofluorescence on coronal sections of E12.5 (**A-C**) and E13.5 (**D-F**), E15.5 (**G-I**) and E18.5 (**J-L**) embryos for Abracl (**A, D, G, J**) and Ki67 (**B, E, H, K**). (**C, F, I** and **L**) are merged images. LGE, lateral ganglionic eminence, MGE, medial ganglionic eminence, VZ, ventricular zone, SVZ, subventricular zone, M, mantle, V-SVZ, ventricular-subventricular zone, STR, striatum. Scale bars: 200µm.

Figure S2. Abracl is coexpressed with the neurogenic factor Ascl1 in the subpallial SVZ. Double immunofluorescence on coronal sections of E12.5 (**A-C**) and E13.5 (**D-F**), E15.5 (**G-I**) and E18.5 (**J-L**) embryos for Abracl (**A, D, G, J**) and Ascl1 (**B, E, H, K**). (**C, F, I** and **L**) are merged images. LGE, lateral ganglionic eminence, MGE, medial ganglionic eminence, VZ, ventricular zone, SVZ, subventricular zone, M, mantle, V-SVZ, ventricular-subventricular zone, STR, striatum. Scale bars: 200µm.

Figure S3. Abracl is coexpressed with Dlx2 in the subpallial SVZ. Double immunofluorescence on coronal sections of E12.5 (**A-C**) and E13.5 (**D-F**), E15.5 (**G-I**) and E18.5 (**J-L**) embryos for Abracl (**A, D, G, J**) and Dlx2 (**B, E, H, K**). (**C, F, I** and **L**) are merged images. LGE, lateral ganglionic eminence, MGE, medial ganglionic eminence, VZ, ventricular zone, SVZ, subventricular zone, M, mantle, V-SVZ, ventricular-subventricular zone, STR, striatum. Scale bars: 200µm.

Figure S4. Abracl is not expressed in the proliferative zones of the pallium. Double immunofluorescence on coronal sections of E12.5 (**A-C**) and E13.5 (**D-F**), E15.5 (**G-I**) and E18.5 (**J-L**) embryos for Abracl (**A, D, G, J**) and Ki67 (**B, E, H, K**). (**C, F, I** and **L**) are merged images. LV, lateral ventricle, CP, cortical plate, Hip, hippocampus. Scale bars: 200µm.

Figure S5. Abracl expression in internal capsule at E15.5 and E18.5. Double immunofluorescence on coronal sections of E15.5 (**A-D**) and E18.5 (**E-H**) embryos for Abracl (**A,E**) and Tubb3 (**B,F**). (**C,G**) DAPI staining of nuclei; tracts are not stained as they lack nuclei, and therefore appear black. (**D**) and (**H**) are merged images. ic, internal capsule. Scale bars: 200µm.

Figure S6. Abracl expression in lateral olfactory tract and anterior commisure. Double immunofluorescence on coronal sections of E15.5 (**A-D**) and E18.5 (**E-H**) embryos for Abracl (**A,E**) and Tubb3 (**B,F**). (**C,G**) DAPI staining of nuclei; tracts are not stained as they lack nuclei, and therefore appear black. (**D**) and (**H**) are merged images. ac, anterior commissure, lot, lateral olfactory tract. Scale bars: 200µm.
