# Supplementary figures and images for: Characterization of the Abracl-Expressing Cell Populations in the Embryonic Mammalian Telencephalon
Source: Biomolecules. 2023 Aug 31;13(9):1337. doi: 10.3390/biom13091337 (PMC10527439; doi:10.3390/biom13091337)

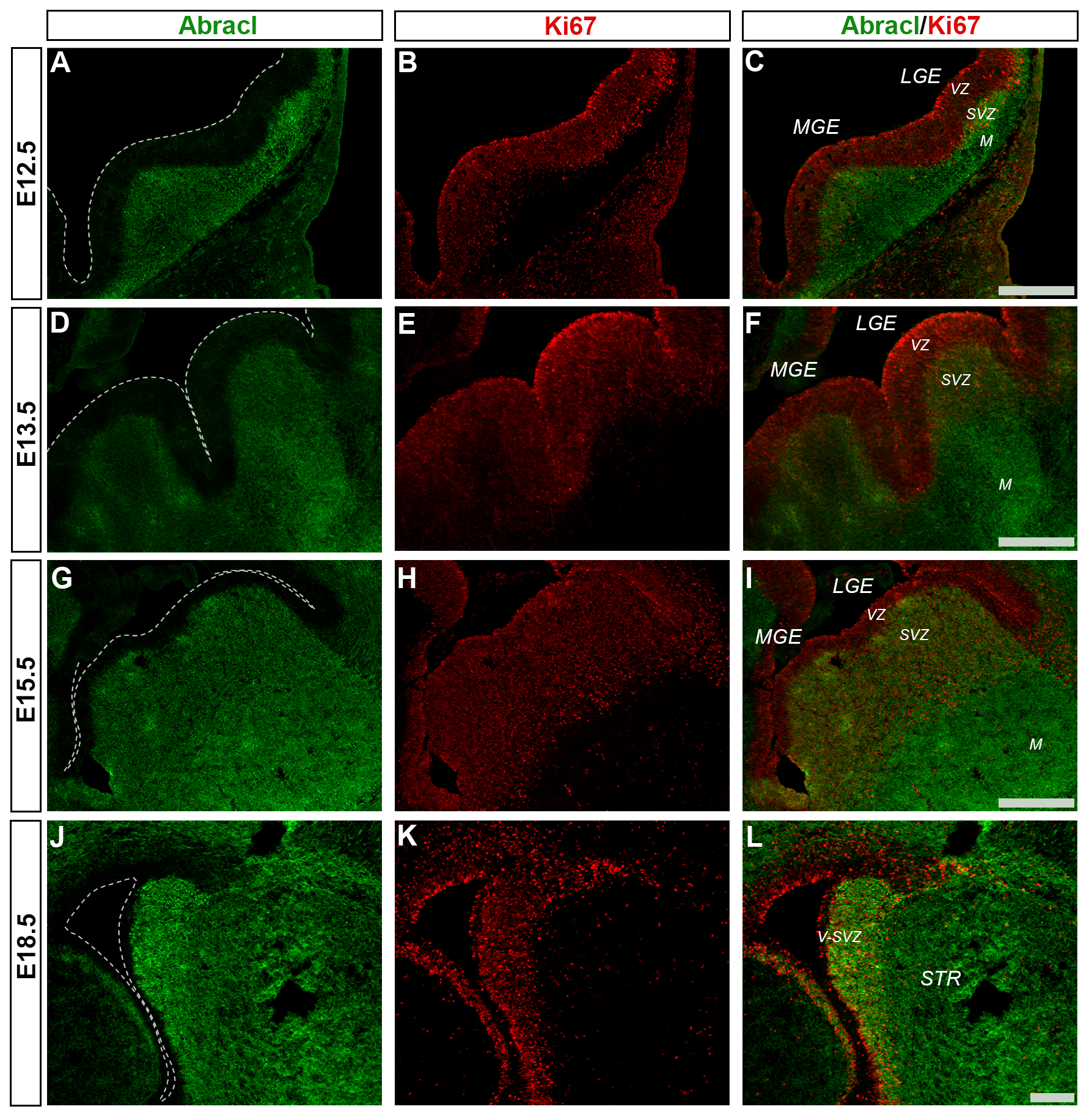

Supplement: Supplementary file 1 [file biomolecules-13-01337-s001.zip › Fig S1 Abracl-ki67 subpallium supp.png]

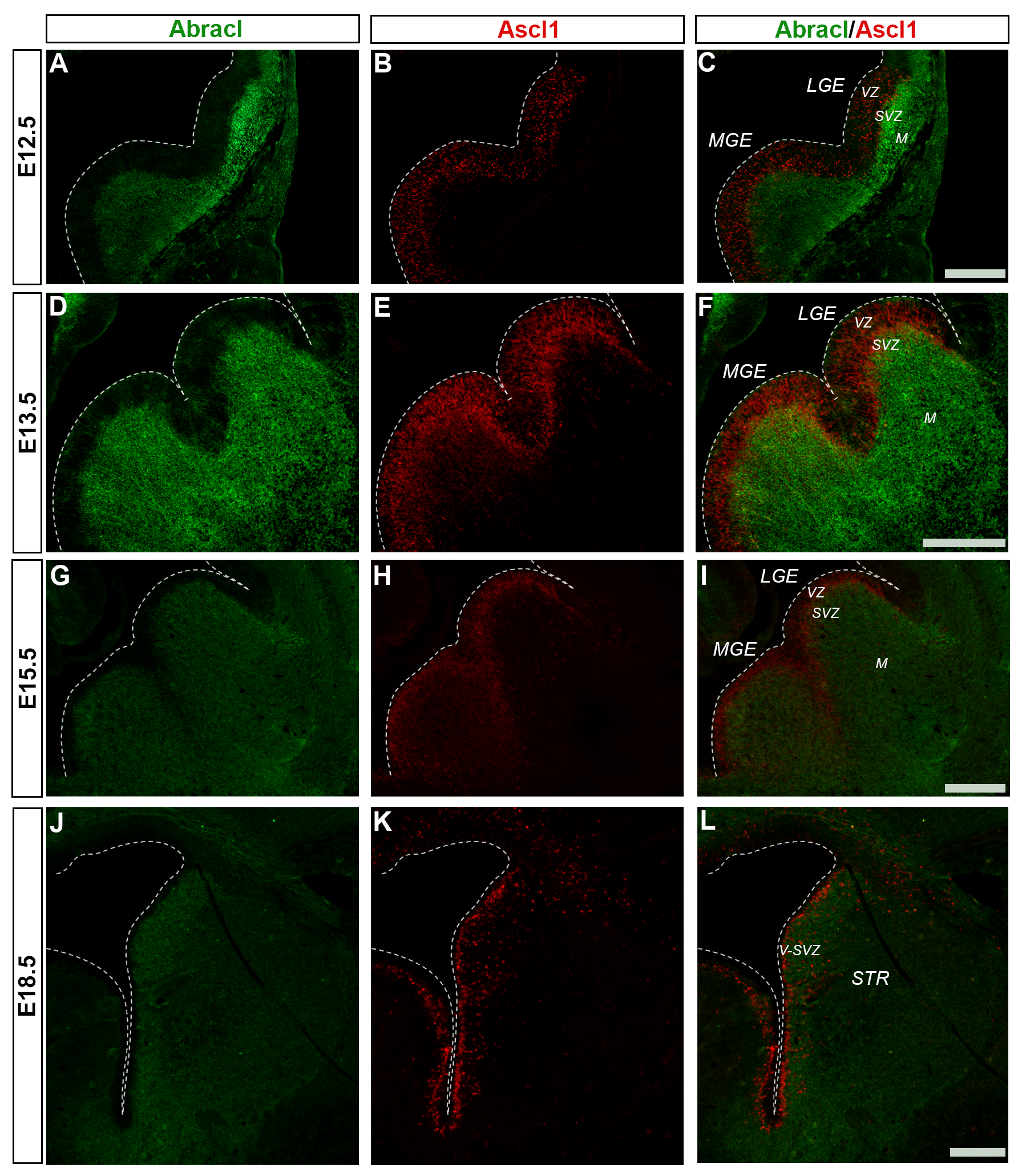

Supplement: Supplementary file 1 [file biomolecules-13-01337-s001.zip › Fig S2 Abracl-ascl1 subpallium supp.png]

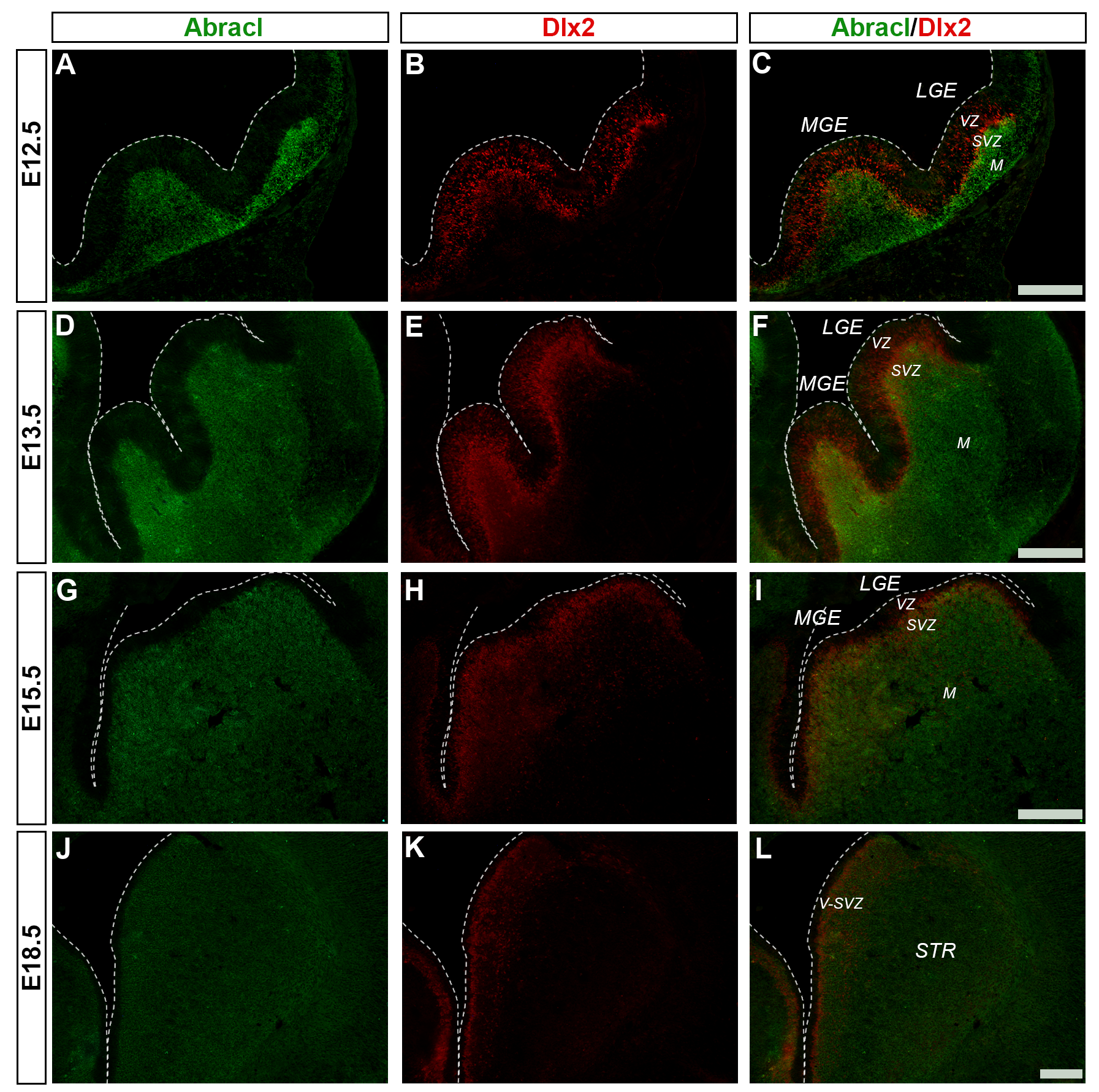

Supplement: Supplementary file 1 [file biomolecules-13-01337-s001.zip › Fig S3 Abracl-dlx2 subpallium supp.png]

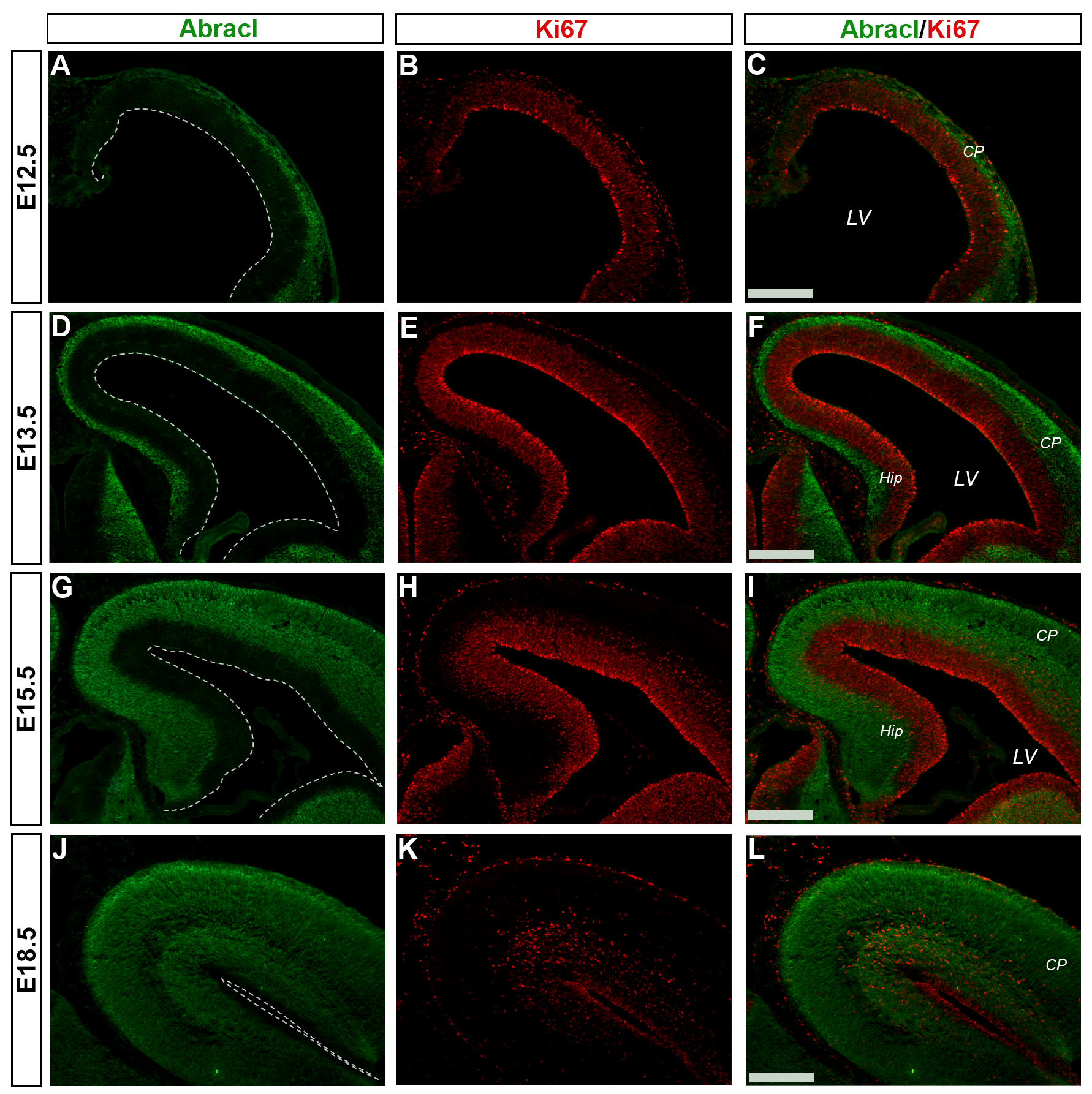

Supplement: Supplementary file 1 [file biomolecules-13-01337-s001.zip › Fig S4 Abracl-Ki67 pallium.png]

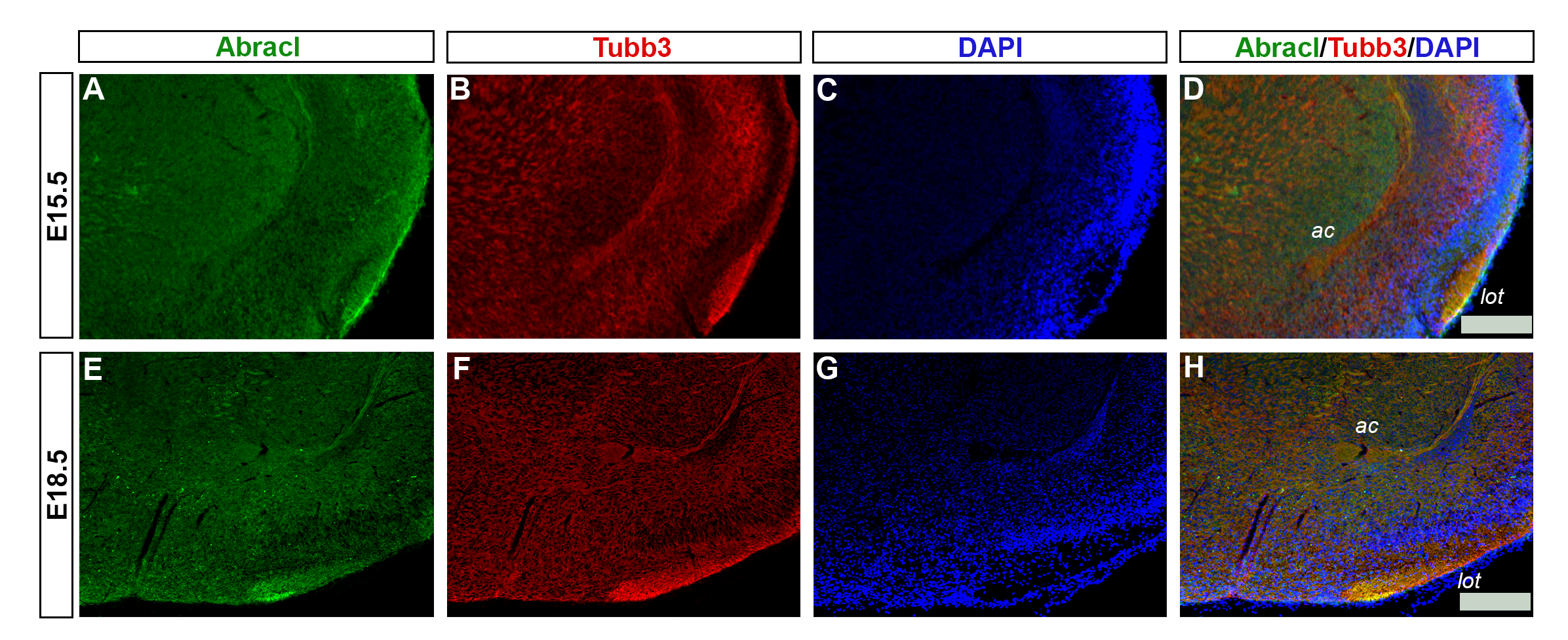

Supplement: Supplementary file 1 [file biomolecules-13-01337-s001.zip › Fig S6 Abracl axons lot flat]
